# The Impact of High Versus Low Sedation Dosing Strategy on Cognitive Dysfunction in Survivors of Intensive Care Units: A Systematic Review and Meta-Analysis

**DOI:** 10.15171/jcvtr.2015.10

**Published:** 2015

**Authors:** Jahan Porhomayon, Philippe Joude, Ghazaleh Adlparvar, Ali A. El-Solh, Nader D. Nader

**Affiliations:** ^1^ VA Western New York Healthcare System, Division of Critical Care Medicine, Department of Anesthesiology, State University of New York at Buffalo School of Medicine and Biomedical Sciences, Buffalo, New York, USA; ^2^ VA Western New York Healthcare System, Division of Pulmonary, Critical Care, and Sleep Medicine, Department of Medicine, State University of New York at Buffalo School of Medicine and Biomedical Sciences, Buffalo, New York, USA; ^3^ Monroe College, State University of New York at Buffalo School of Medicine and Biomedical Sciences, Rochester, New York, USA; ^4^ VA Western New York Healthcare System, Division of Cardiothoracic Anesthesia and Pain Medicine, Department of Anesthesiology, State University of New York at Buffalo School of Medicine and Biomedical Sciences, Buffalo, New York, USA

**Keywords:** Posttraumatic Stress Disorder, Depression, Cognitive Function, Sedation, Delirium, Cognitive Function

## Abstract

***Background:*** The practice of low vs. high sedation dosing strategy may impact the cognitive and mental health function in the intensive care unit (ICU). We aim to demonstrate that high sedation strategy will result in change of mental health function in ICU patients.

***Methods:*** We performed a systemic search and meta-analysis of medical databases in MEDLINE (from 1966 to March 2013) and EMBASE (from 1980 to March 2013), as well as the Cochrane Library using the MESH terms "Intensive Care Unit," and "Mental Health, for assessing the impact of sedation on posttraumatic stress disorder (PTSD) or anxiety/depression and delirium in the mix ICU setting including cardiac surgery patients. A total of 1216 patients were included in the final analysis.

***Results:*** We included 11 studies in the final analysis and concluded that high dose sedation strategy resulted in higher incidence of cognitive dysfunction with *P* value of 0.009. The result for subgroup of delirium showed *P* = 0.11 and PTSD/depression or anxiety of P = 0.001, Heterogeneity I2 was 64%. Overall analysis was statistically significant with a *P* value of 0.002.

***Conclusion:*** High sedation dosing strategy will negatively affect cognitive function in critically ill patients. Large randomized trials are needed to address cognitive dysfunction in subgroup of patients with delirium.

## Introduction

The impact of posttraumatic stress disorders (PTSD), depression or anxiety, and delirium in the intensive care unit (ICU) are profound and patients are at increased exposure for cognitive dysfunction.^1,2^ There are various predisposing etiologies for the development of PTSD,^3^ agitation or depression and delirium in critically ill patients. One intervention that has received special attention in the ICUs is the practice of sedation and its influence for the development of PTSD/depression and delirium. Early ICU practices relied on utilizing heavy sedation with subsequent recognition of increased delirium and its associated higher morbidity and mortality. Additionally, recognition about PTSD started around 1992 when the World Health Organization (WHO) initially introduced the classification and it was later supplemented in the DSM-IV edition.^4^ In the DSM-V, the diagnosis of PTSD has undergone multiple changes. These changes include shifting PTSD placement from within the anxiety disorders into a new category of traumatic and stressor-related disorders, alterations in the definition of a traumatic event, shifting of the symptom cluster structure from three to four clusters, the addition of new symptoms including persistent negative beliefs and expectations about oneself or the world, persistent distorted blame of self or others, persistent negative trauma related emotions, risky or reckless behaviors, and the addition of a dissociative states specified.^5^

The term sedation as defined by the American Society of Anesthesiology comprises continuum of states ranging from minimal sedation (anxiolysis) through general anesthesia.^6^ The practice of intensive care sedation for critically ill patients has evolved for many decades from an era of liberal use of sedatives to a much more restricted use. One critical and detrimental aspect of the use of sedation is its impact on mental health function. Over the last few decades, investigators have raised concerns over the long term effect of sedative use on mental health and cognitive function.^7,8^ Several randomized trials have especially looked at the occurrence of PTSD, anxiety/depression and delirium in ICU population.^9,10^ Recently, trials have also been conducted to evaluate the impact of high dose sedation on mortality. It is now established that lighter sedation strategy is beneficial to the critically ill patient.

We believe it is necessary to emphasize the positive aspect of light sedation strategy and its outcome on cognitive dysfunction. The risk factors and impact of other etiologies for cognitive dysfunction in ICU has been clearly defined in previous studies but to our knowledge no previous studies on sedation dosing strategy has ever been completed. The aim of this study was to assess the relevant results on the impact of heavy vs. light sedation dosing strategy on cognitive function.

## Methods

**Figure 1:**
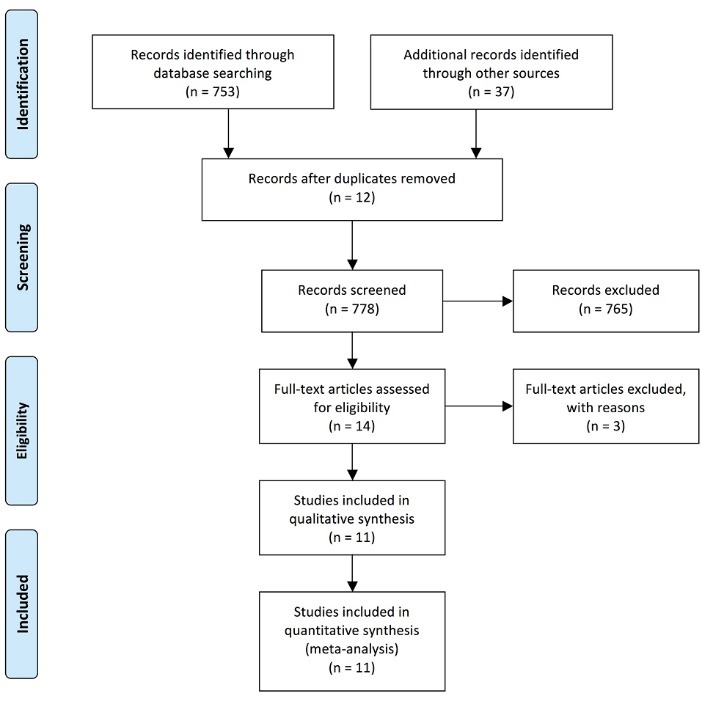
Flow diagram for inclusion and exclusion of the studies

We searched the electronic databases MEDLINE (from 1966 to March 2013) and EMBASE (from 1980 to March 2013), as well as the Cochrane Library using the MESH terms “Intensive Care Unit,” and “Mental Health”. We further narrowed the search by using the filters “post-traumatic stress disorders, “sedation,” “delirium” and “anxiety or depression”. Furthermore, we reviewed reference lists of original and reviewed articles to search for more studies. Only those studies that were published as full-length articles were considered. No language restriction was applied. When confronted with different time interval for evaluation of cognitive dysfunction, months after discharge was considered for final analysis rather than evaluation at the time of discharge from ICU (Figure 1). After careful examination of titles and abstracts, a total of 753 citations were identified from databases. Additional 37 articles were identified through search from other databases and from the references of 753 articles. Twelve records were excluded for duplication or repetition. 778 records were screened. Narrowing the search for delirium and PTSD/depression or agitation yielded 227 articles. Two authors evaluated 227 articles independently and 14 articles were included for analysis. One prospective trial and two retrospective studies were excluded because sedation scores were not reported. Furthermore, we also reviewed the reference lists of the selected 10 studies to search for additional trials. Finally, 10 prospective randomized controlled trials (RCTs) and one observational study were identified for analysis (Figure 1).

Deep sedation was defined as: either the patient had no response to voice, movement or eye opening with physical stimulation or unarousable. Light sedation was defined as: patient not fully alert, but has sustained awakening to voice with possibility of restlessness or agitation.

### Inclusion and Exclusion Criteria

For inclusion, studies had to fulfill the following criteria: (*a*) original article with a sample size of five or more participants, (*b*) adult population, (*c*) evaluation of sedation by accepted standard scoring system, and (*d*) evaluation of PTSD or anxiety/stress/depression and delirium by a validated standard method. We excluded evaluation after discharge from ICU, since long term outcome was our target of interest. Articles were excluded if sedation scores were not reported. We also excluded studies involving central nervous system injury, stroke, neuro-intensive care unit, and neuro-trauma ICUs. We also excluded studies with patients having preexisting psychological disorders, alcohol abuse, PTSD, depression and anxiety disorders. If multiple published reports from the same study were available, we included only the one with the most detailed information for both exposure and outcome (Table 1 and 2).

**Table 1:** The List of Studies Pairing Sedation Scores With PTSD/Depression or Anxiety

**Author**	**Design**	**Setting**	**Number**	**Control**	**Intervention**
Kress et al^30^	Retrospective	MICU	32	Standard sedation (heavy) vs. daily sedation interruption (light) based on RASS score	PTSD or anxiety/depression
Jackson et al^8^	Prospective	MICU	80	Standard sedation (heavy) vs. sedation protocol paired with weaning trial (light) based on RASS score	PTSD or anxiety/depression
Strøm et al^35^	Prospective	Mix ICU	26	Standard sedation (heavy) vs. no sedation except haloperidol (light) based on RASS score	PTSD or anxiety/depression
Treggiari et al^9^	Prospective	Mix ICU	102	Standard sedation (heavy) vs. sedation protocol paired with weaning trial (light) based on RASS score	PTSD or anxiety/depression

Abbreviations: ICU, intensive care unit; MICU, medical intensive care unit; PTSD, posttraumatic stress disorder; RASS, richmond agitation sedation score.

**Table 2:** The List of Studies Pairing Sedation Scores Delirium.

**Author**	**Design**	**Setting**	**Number**	**Control**	**Intervention**
Aydogan et al^27^	Prospective	PACU	42	Sedation with either DEX (light) or MDZ (heavy) based on RASS score	Delirium assessment
Colombo et al^29^	Prospective	MICU	314	Standard sedation protocol (heavy) vs. lighter sedation strategy with reorientation strategy	Delirium assessment
Girard et al^33^	Prospective	MICU	168	Standard sedation (heavy) vs. sedation protocol paired with weaning trial (light) based on RASS score	Delirium for secondary outcome
Ruokonen et al^32^	Prospective	MICU SICU	12	Sedation with either DEX (light) or MDZ (heavy) based on RASS score	Delirium for secondary outcome
Shehabi et al^31^	Prospective	MICU	37	Sedation with either DEX (light) or MDZ (heavy) based on RASS Score	Delirium for secondary outcome
Strom et al^37^	Prospective	ICU	113	Sedation group with MDZ or propofol or no sedation group	Delirium for secondary outcome
Brown et al^36^	Prospective	PACU	84	Deep vs. low sedation utilizing bispectral index monitor	Delirium /survival

Abbreviations: MDZ, midazolam; RASS, richmond agitation sedation score; DEX, dexmedetomidine.

### Data Extraction

Articles were identified in a staged process whereby titles were initially screened for potential eligibility. Abstracts and full texts of those potentially eligible were assessed by two reviewers (PA and JP) independently and any disagreements were resolved by consensus. Data included the first author’s surname, year of publication, country of origin of the population studied, study design, number of participants, participants’ age, gender, and modalities used for sedation or PTSD/anxiety/depression or delirium assessment. Whenever needed, we obtained additional information about a specific study by directly contacting the primary author.

### Statistical Analysis

The Cochrane collaborative review for systemic analysis (RevMan software 5.2) was used to calculate the risk ratio for the incidence of mental health dysfunction for each study. I^2^ was used to assess statistical heterogeneity with a value below 30% standing for low heterogeneity, a value between 30% and 60% standing for moderate heterogeneity and a value above 60% standing for high heterogeneity. Fixed effect model was used in all analyses. Risk bias was assessed according to Cochrane review. A *P* value of 0.1 or less was significant.

## Results

### Meta-analysis of Sedation Practices on Cognitive Dysfunction Outcome

**Figure 2:**
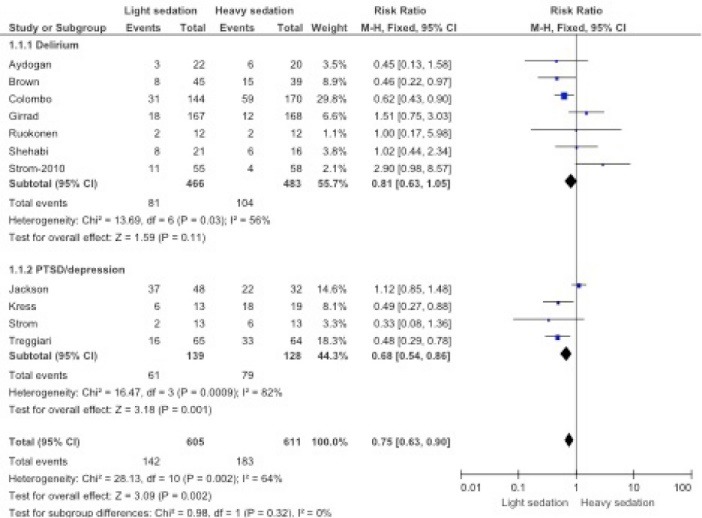
Forest Plot of Comparison: 1 Postcognitive Dysfunction, Outcome: 1.1 High vs. Low-dose Sedation.

**Figure 3:**
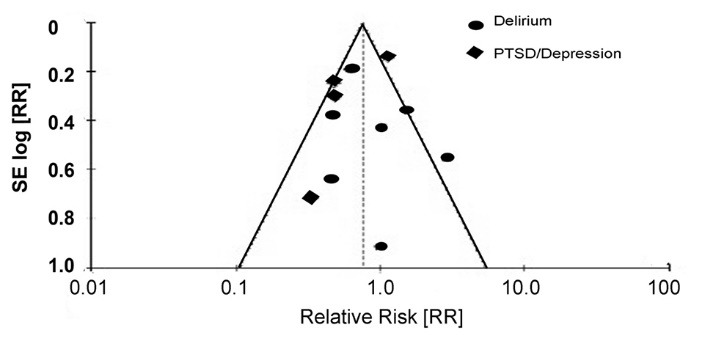
Funnel Plot of 11 Studies Including Delirium and PTSD /Depression or Anxiety.

We included 10 randomized controlled studies and 1 retrospective study for impact of sedation level on cognitive function. A total of 1216 participants were included with (M-H, Fixed 95% CI) and effect estimate 0.75 (0.63, 0.90) in statistical analysis. Heterogeneity was 64%, Chi² = 28.3, df = 10, (*P* = 0.002). Total overall effect or Z score of 3.09 with *P *= 0.002. The incidence of cognitive dysfunction was reported as 142/605 (23%) with the use of lower sedation scores and 183/611 (29%) with the use of higher sedation scores. There were 6 studies favoring cognitive dysfunction in high sedation group, 3 studies favoring cognitive dysfunction in low sedation group and 2 studies were neutral. Overall, higher sedation was associated with higher incidence of cognitive dysfunction with *P* value of 0.002. Subgroup difference was not significant with Chi² = 0.89, df=1, (*P* = 0.32) and I^2^ of zero (Figure 2 and 3).

### Meta-analysis of Subgroup for Sedation Practices on PTSD/Depression Outcome

We included 3 randomized controlled studies and one retrospective study for impact of sedation level on PTSD/anxiety or depression. A total of 267 participants were included with (M-H, Fixed 95% CI) and effect estimate 0.68 (0.54, 0.86) in statistical analysis. Heterogeneity was 82%, Chi² = 16.47, df = 3 (*P* = 0.0009). Total overall effect or Z score of 3.18 with *P *= 0.001. The incidence of PTSD/depression or anxiety was reported as 61/139 (43%) with the use of lower sedation scores and 79/128 (61%) with the use of higher sedation scores. There were 3 studies favoring PTSD in high sedation group and one study favoring cognitive dysfunction in low sedation group. Overall, higher sedation was associated with higher incidence of mental dysfunction (Figure 2).

### Meta-analysis of Subgroup for Sedation Practices on Delirium Outcome

We included 7 randomized controlled studies for impact of sedation on delirium. A total of 949 participants were included with risk ratio (M-H, Fixed, 95% CI) and effect estimate of 0.81 (0.63, 1.05) in statistical analysis. Heterogeneity was 56%, Chi² = 13.69, df = 6 (*P* = 0.03). Total overall effect or Z score of 1.59 with *P *= 0.11. The incidence of delirium was reported as 81/466 (17%) with the use of lower sedation scores and 104/483 (22%) with the use of higher sedation scores. There were 3 studies favoring delirium with higher sedation scores, and 2 studies favored delirium in lower sedation group and two studies were neutral. Overall, higher sedation level was associated with higher incidence of delirium with *P* value of 0.11 (Figure 2 and 3).

Assessment of risk of bias was low for all RCTs and intermediate for one retrospective study. Missing data was appropriately outlined in 8 trials and not well delineated in 3 trials. Data analysis was according to trials guidelines and well documented in all trials. Sensitivity analysis and combined risk estimates did not materially change the sensitivity analyses.

## Discussion

Despite the overwhelming evidence of beneficial effects of lighter sedation strategy, many ICUs still continue to use high dose sedation practices for managing patient on mechanical ventilation.^11,12^ The current standard dictates that ICUs around the globe should use either sedation holiday^13,14^ or protocol based sedation strategy for pairing sedation to spontaneous weaning from ventilation trials.^15,16^ Nevertheless, the recent publication by Burry and colleagues, an observational study of 51 ICUs revealed that sedation and pain assessment tools were used only in 53% and 19% of cases respectively.^17^ Furthermore, the impact of sedation on PTSD, anxiety/depression and delirium has been evaluated previously in RCTs and several retrospective trials.^18^ The study by Brummel et al ^19^ indicated that longer delirium duration in ICU was independently associated with increased odds of disability in activities of daily living and worse motor or sensory function after discharge from hospital. Wolters et al study also reported that ICU survivors had increased cognitive dysfunction after discharge from ICU.^20^ Deep and light sedation strategies were discussed in this analysis with the assessment of validated sedations scores. PTSD, depression/anxiety and delirium were evaluated by various tools and scoring system.^21^ Depression is usually associated with PTSD and can also be evaluated by scoring system such as the hospital anxiety and depression scale (HADS),^22^ Beck depression scale^23^ and the impact of events scale (IES).^24^ PTSD was evaluated by impact event scale (IES), posttraumatic stress syndrome 10-questions inventory (PTSS-10), a self-report scale for the diagnosis of PTSD,^25^ Revised IES that evaluates signs of PTSD.^26^ Delirium was assessed with Confusion assessment method in ICU. These are all standard, generic measures used widely in the assessment of cognitive function. In this study we evaluated 10 RCTs and one retrospective analysis utilizing a traditional method of sedation compared to protocol directed sedation strategy. Since our primary aim was to assess the long-term impact of heavier sedation on cognitive function, we excluded events on time of discharge from ICUs.

In general, several well conducted high quality prospective trials were presented in this article.^9,27-30^ The study by Shehabi and colleagues specifically looked at the first 48 hours of admission on mechanical ventilation in the ICU in 250 patients.^31^ He demonstrated that in the first 48 hours, sedation was heavier with higher incidence of confusion and delirium. However, Shehabi et al analysis did not show differences in the subsequent incidence of delirium or agitation during lighter sedation. The study by Roukonen et al^32^ could not show a difference in cognitive dysfunction between dexmedetomidine and midazolam or propofol group. Girard et al^33^ and Strom et al^34^ conducted a well-respected and well-designed randomized control trials and both concluded that either a wake up and breathe protocol that pairs daily spontaneous awakening trials with daily spontaneous breathing trials or an strategy of zero to minimal sedation resulted in better outcomes and less delirium for mechanically ventilated patients in intensive care. The long-term effect of psychological outcome and PTSD was evaluated by Strom et al^35^ and indicated higher PTSD related symptoms in heavy sedation group.

In all these studies, sedation practices varied across the different intensive care units or recovery rooms. Sedation interruptions were mostly utilized and different sedative agents were used. The final goal was to have an awake and alert patient who could perform weaning trials according to each respected ICU protocols. Therefore, clinical heterogeneity was moderate in this analysis. Statistical heterogeneity was 82% for PTSD/depression or anxiety and 56% for delirium respectively for subgroup but when sub-group were combined heterogeneity decreased to 64%. The study by Samuelson et al was excluded. In Samuelson et al study the number of patients in light vs. heavy sedation group was not clearly documented but according to author the PTSD group had higher anxiety scores in heavier sedated group. The study by Brown and colleagues^36^ was very interesting since he evaluated sedation level by bispectral index monitor. His group concluded that the incidence of delirium was higher in the heavy sedation group (19% vs. 40%; *P* = 0.02) but mortality and survival after one year was only significant in patients with higher comorbidities. An editorial commentary suggested larger randomized trials are needed to assess cognitive outcome and mortality.^37^

The current individualized studies do not have adequate power size to provide clinicians with a reliable recommendation for the impact of sedation practices in ICUs and its impact on cognitive function. However, when we combined all studies, we were able to demonstrate in our Forest plot (Figure 2) a close association between cognitive dysfunction and higher sedation score. This association was less visible in the delirium group with *P* = 0.11 but highly significant in PTSD group (Figure 2).

The Society of Critical Care Medicine updated its sedation and pain management guidelines in 2014. The new recommendation emphasizes the concept of analgesic-sedative therapy where pain assessment takes priority over sedation assessment. This is particularly important in trauma and surgical ICUs where many patients present with acute pain from surgical incisions or fractures. We hope that with implementation of such strategy, we will decrease excessive reliance on sedative agents and at the same time decrease the incidence of PTSD or agitation and delirium.

In summary, higher sedation dosing strategy will impact cognitive function in critically ill patients both medically and psychologically. Larger randomized prospective trials are needed to better address the long-term effect of sedative agents as well as sedation practices in the ICU and its impact on cognitive function. Newer and emerging therapies with newer antipsychotic or sedative agents are promising but still insufficient data exist to make any solid recommendations to change current practice.

## Ethical Issues

Not applicable

## Competing Interests

The authors declare that they have no conflict of interests.
